# Supplementary material for: Do ‘leaders’ in change sound different from ‘laggers’? The perceptual similarity of New Zealand English voices
Source: PLoS One. 2025 Dec 11;20(12):e0338199. doi: 10.1371/journal.pone.0338199 (PMC12697940; doi:10.1371/journal.pone.0338199)
Supplement: Attachment — Submitted filename: PLOS_ResponseToReviewers.docx [file pone.0338199.s002.docx]

# Response to reviewers and editors

**We sincerely thank the reviewers for their time spent reviewing the revised manuscript. The following document lists all comments and feedback, with our responses in bold.**

**Comments to the Author**

1. If the authors have adequately addressed your comments raised in a previous round of review and you feel that this manuscript is now acceptable for publication, you may indicate that here to bypass the “Comments to the Author” section, enter your conflict of interest statement in the “Confidential to Editor” section, and submit your "Accept" recommendation.

Reviewer #1: All comments have been addressed

Reviewer #3: All comments have been addressed

2. Is the manuscript technically sound, and do the data support the conclusions?

The manuscript must describe a technically sound piece of scientific research with data that supports the conclusions. Experiments must have been conducted rigorously, with appropriate controls, replication, and sample sizes. The conclusions must be drawn appropriately based on the data presented.

Reviewer #1: Yes

Reviewer #3: Yes

3. Has the statistical analysis been performed appropriately and rigorously?

Reviewer #1: Yes

Reviewer #3: Yes

4. Have the authors made all data underlying the findings in their manuscript fully available?

The [PLOS Data policy](http://track.editorialmanager.com/CL0/http:%2F%2Fwww.plosone.org%2Fstatic%2Fpolicies.action%23sharing/1/010f01995ff66ed7-66d044ad-1896-4c3c-b2b0-926744c02714-000000/ogrXp78LHpA182__nzlhSXBQebGpUvjk0mG72-9L3uE=229) requires authors to make all data underlying the findings described in their manuscript fully available without restriction, with rare exception (please refer to the Data Availability Statement in the manuscript PDF file). The data should be provided as part of the manuscript or its supporting information, or deposited to a public repository. For example, in addition to summary statistics, the data points behind means, medians and variance measures should be available. If there are restrictions on publicly sharing data—e.g. participant privacy or use of data from a third party—those must be specified.

Reviewer #1: Yes

Reviewer #3: Yes

5. Is the manuscript presented in an intelligible fashion and written in standard English?

PLOS ONE does not copyedit accepted manuscripts, so the language in submitted articles must be clear, correct, and unambiguous. Any typographical or grammatical errors should be corrected at revision, so please note any specific errors here.

Reviewer #1: Yes

Reviewer #3: Yes

6. Review Comments to the Author

Please use the space provided to explain your answers to the questions above. You may also include additional comments for the author, including concerns about dual publication, research ethics, or publication ethics. (Please upload your review as an attachment if it exceeds 20,000 characters)

Reviewer #1: The authors have addressed all substantive reviewer concerns and significantly strengthened the paper. With minor refinements to figures and a forward-looking discussion of sample size/power issues, this manuscript will be ready for publication.

**In response to this feedback, we have integrated a discussion of statistical power and sample sizes into our ‘Limitations’ section of the manuscript. We specifically state:**

**“Finally, we would like to discuss statistical power. We did not conduct a power analysis to determine the desired participant sample size because dimension-reduction techniques such as our pre-registered MDS analysis do not test a null hypothesis. MDS does not, by extension, produce the Type I or Type II statistical errors power analyses are intended to mitigate [103]. The extent to which larger listener sample sizes improve the fit (i.e., stress) of an MDS analysis is, nonetheless, a relevant and underexplored question. Rodgers [106] found samples as small as one to six participants can provide good Metrix Recovery of original distances and comparable stress values to larger samples, and our sample size exceeds both those numbers and common participant numbers applications of MDS in psychology [see 104] and linguistics [e.g., 78, 80, 105]. Discussions of stress and “sample size” in applications of MDS otherwise focus on the number of input items [e.g., 104, 107], which in our case was constrained by QuakeBox participant demographics. The exact relationship between our participant sample size and MDS fit remains, however, an open question.**

**As the question of pairwise ratings emerged as our analysis progressed, we did not pre-register the reported GAMM analysis or conduct a post-hoc power analysis of the participant sample size. The general risk of post-hoc power analyses aside [see 108, 109], there is limited precedent for determining participant sample sizes in the application of GAMMs. Linguistic papers that discuss GAMMs and statistical power consider power across model types or in the model-fitting process [e.g., 110, 111], rather than in relation to sample sizes and Type I/II error probability. Furthermore, simulation methods have been developed for assessing statistical power for generalised linear mixed models [e.g., 112, 113], but they do not currently apply to the outputs of GAMM models in R. In other words, there is not yet an accessible, conventionalised, approach to informing a desired sample sizes for fitting GAMMs in linguistics. As such, we highlight simulation-based approaches to participant sampling in both MDS and GAMMs as potential directions of future research and methodological innovation.”**

**We have re-generated the figures to align with PLOS One’s requirements and run them through the required PACE tool. We have also adjusted Figure 2 to be more readable in terms of font and legend sizes and updated the formatting of the manuscript text and tables.**

Reviewer #3: I read the ms a few times. The authors did a great job revising the manuscript. The paper's structure became clearer, which improved its readability. Also, the methodological choices are well explained. I have no further questions.

**We thank the reviewer for their kind comments.**
